# Highly efficient transgene‐free targeted mutagenesis and single‐stranded oligodeoxynucleotide‐mediated precise knock‐in in the industrial microalga *Euglena gracilis* using Cas9 ribonucleoproteins

**DOI:** 10.1111/pbi.13174

**Published:** 2019-06-11

**Authors:** Toshihisa Nomura, Komaki Inoue, Yukiko Uehara‐Yamaguchi, Koji Yamada, Osamu Iwata, Kengo Suzuki, Keiichi Mochida

**Affiliations:** ^1^ RIKEN Center for Sustainable Resource Science Tsurumi‐ku, Yokohama Japan; ^2^ RIKEN Baton Zone Program Tsurumi‐ku, Yokohama Japan; ^3^ euglena Co., Ltd. Tokyo Japan; ^4^ Kihara Institute for Biological Research Yokohama City University Totsuka‐ku, Yokohama Japan; ^5^ Graduate School of Nanobioscience Yokohama City University Tsurumi‐ku, Yokohama Japan; ^6^ Institute of Plant Science and Resources Okayama University Kurashiki, Okayama Japan

**Keywords:** *Euglena gracilis*, Microalgae, genome editing, ribonucleoproteins, CRISPR/Cas9, transgene free, knock‐in, single‐stranded oligodeoxynucleotides

Clustered regularly interspaced short palindromic repeat (CRISPR)/CRISPR‐associated nuclease 9 (Cas9) system‐based genome editing has been applied to a wide range of species, including economically important agricultural crops (Doudna and Charpentier, 2014). Genome editing by the direct delivery of ribonucleoproteins (RNPs) offers various advantages compared to the conventional transgene‐based method, including high efficiency, low time requirements, reduced off‐target effects and low cytotoxicity (Jeon *et al*., 2017; Spicer and Molnar, 2018). Moreover, transgene‐free genome‐edited organisms may bypass current regulations for genetically modified organisms (Jeon *et al*., 2017; Spicer and Molnar, 2018), which is suitable for food and healthcare applications such as the molecular breeding of crops and microalgae. However, owing to the low mutation efficiency of screening‐less and DNA‐free RNP‐based genome editing methods in microalgae (~1%) (Jeon *et al*., 2017; Spicer and Molnar, 2018), transgene‐free genome editing technology needs to be further improved for practical applications.

*Euglena gracilis*, a unicellular photosynthesizing flagellate, is an industrially exploited microalga. *E. gracilis* is rich in nutrients and accumulates a crystallized β‐1,3‐glucan, paramylon (Inui *et al*., 2017), which has various bioactive functionalities (Suzuki, 2017). Therefore, mass‐cultured *E. gracilis* is a commercial source for functional foods, feeds and cosmetics (Suzuki, 2017). Moreover, under anaerobic conditions, paramylon is degraded and converted to wax esters, which mainly consist of myristic acid (C14:0) and myristyl alcohol (C14:0) (Inui *et al*., 2017). As the wax ester of *E. gracilis* is easily reformed to a biofuel having a low freezing point, it is suitable as a source for biojet fuel (Inui *et al*., 2017). Despite these promising features of *E. gracilis* as a renewable resource, the establishment of its effective targeted mutagenesis has been a long‐standing challenge. In this study, we developed a highly efficient transgene‐free targeted mutagenesis and single‐stranded oligodeoxynucleotide (ssODN)‐mediated knock‐in in *E. gracilis* using Cas9 RNPs, which provides the first evidence that *E. gracilis* is a genome‐editable organism.

To establish a transgene‐free mutagenesis method based on Cas9 RNPs in *E. gracilis*, we designed two target sequences within the second exon of its *glucan synthase‐like 2* (*EgGSL2*, GenBank accession number: LC225615) gene (Figure 1a), which is putatively involved in paramylon biosynthesis (Tanaka *et al*., 2017). Each crRNA and tracrRNA was synthesized by Alt‐R CRISPR‐Cas9 system in Integrated DNA Technologies (IDT), and the same amounts of 100 μm solutions were used for annealing. Cas9 RNP complexes were prepared by mixing the cooled gRNA complex and Alt‐R S.p. Cas9 Nuclease V3 (IDT, 62 μm solution) at a ratio of 3:2 (v/v) and incubating for 15 min at 20–25°C. Electroporation solution was prepared by mixing CM medium (pH 5.5) without sodium citrate and a filter‐sterilized 0.3 m sucrose solution at a ratio of 3:2 (v/v). *Euglena gracilis* Z strain provided by IAM (Tokyo, Japan) was cultured in KH medium (pH 5.5) on a rotary shaker (120 rpm) at 28°C under 50 μmol continuous light for 3 days, and 1 mL of the culture liquid was centrifuged at 400 × *g* for 30 s. *Euglena gracilis* pellets were washed once with electroporation solution and resuspended with electroporation solution at a concentration of 1 × 10^6^ cells/mL. For electroporation, 2 μL of RNP complex solution was added to 48 μL of *E. gracilis* suspension. We introduced Cas9 RNPs for the target regions in *EgGSL2* into *E. gracilis* cells by electroporation using a NEPA21 Super Electroporator with a 2‐mm gap cuvette EC‐002 (NEPPA GENE). Immediately after electroporation, 1 mL of KH medium was added, followed by transfer to a 12‐well plate and culture on a rotary shaker (120 rpm) at 28°C under dark conditions (Figure 1b). After culture for 72 h, we confirmed that the *E. gracilis* cells exhibited fewer but larger paramylon granules with these two *EgGSL2*‐targeting RNPs (Figure 1c). The average frequencies of altered phenotypes for targets 1 and 2 were 71.3% and 80.6%, respectively (Figure 1d). We amplified the *EgGSL2* locus using Gflex polymerase (Takara Bio) and detected mutagenesis by a T7 endonuclease I assay using the Alt‐R Genome Editing Detection Kit (IDT) at 72 h after the direct delivery of Cas9 RNPs (Figure 1e). We also performed Sanger sequencing of both the target regions and identified various insertion and deletion (indel) patterns (Figure 1f). Based on amplicon sequencing of *EgGSL2* target regions amplified with KAPA HiFi HS ReadyMix (KAPA Biosystems) using an ion proton system (Thermo Fisher Scientific), we detected indel mutation rates of 77.7–86.8% and 88.8–90.1% at *EgGSL2* targets 1 and 2, respectively, (Figure 1g) at 72 h after electroporation as assessed by the Cas‐Analyzer software (Park *et al*., 2017). Additionally, the simultaneous introduction of two Cas9 RNPs targeting different sites, targets 1 and 2, into *EgGSL2* yielded an approximately 200‐bp deletion between the targets (Figure 1h,i), suggesting the ability to generate long deletion mutants of targeted regions in *E. gracilis*.

**Figure 1:**
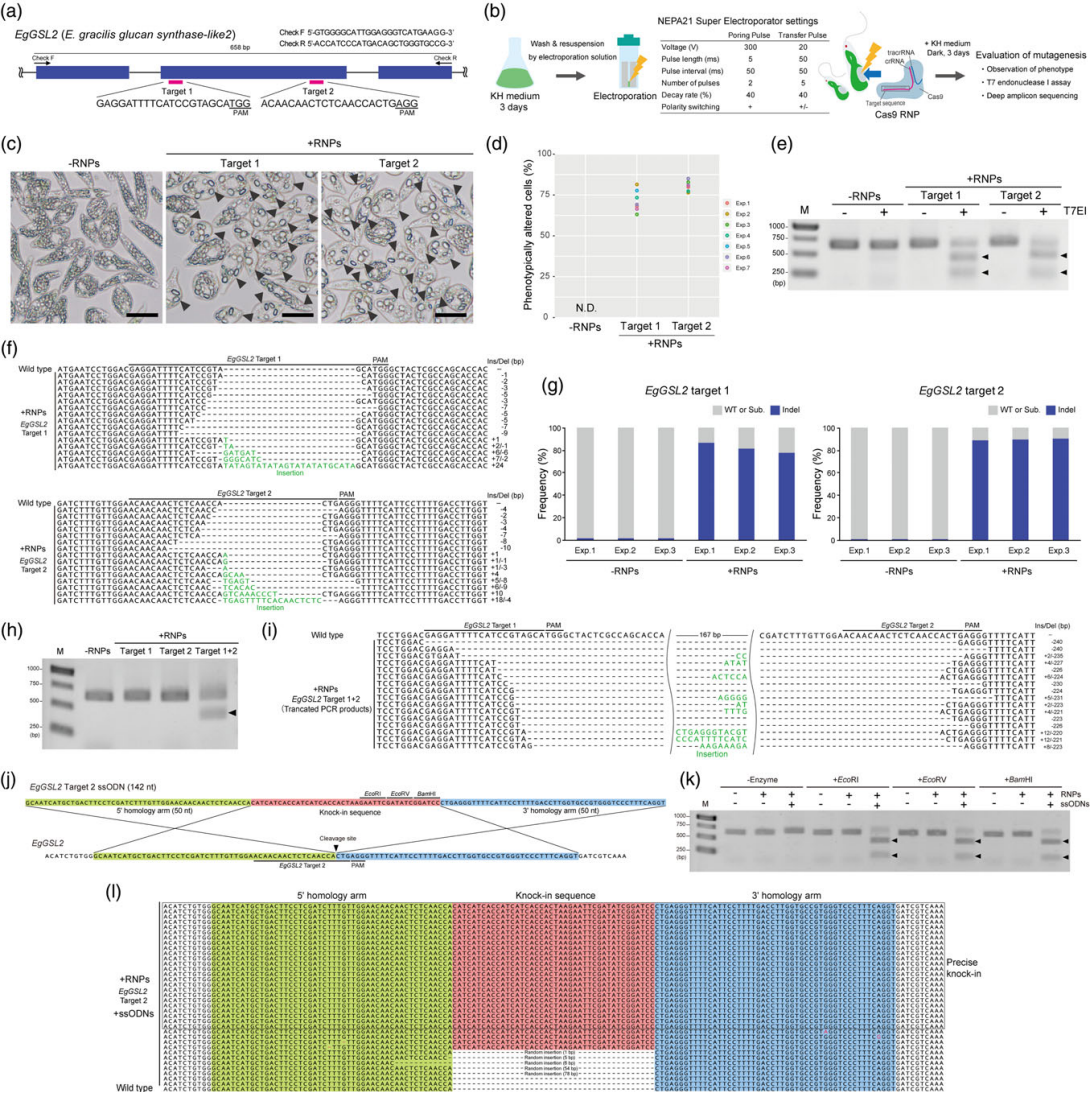
Highly efficient targeted mutagenesis and ssODN‐mediated knock‐in by Cas9 RNPs in *Euglena gracilis*. (a) Schematic illustration of two targeting sites and primer sequences on the partial genomic sequence of the 5′ end of the *EgGSL2* gene. (b) Experimental workflow and settings for electroporation of the targeted mutagenesis methods using Cas9 RNPs in *E. gracilis*. (c) Representative images of *E. gracilis* cells untreated with RNP (‐RNPs) and those treated with RNP targeting the *EgGSL2* genes at 72 h after electroporation. Scale bars = 25 μm. Arrowheads indicate *E. gracilis* cells with large paramylon granules. (d) Percentage of phenotypically altered cells at 72 h after electroporation in ‐RNPs and cells treated with RNP targeting the *EgGSL2* gene. The graph represents the results of seven independent experiments (Exp. 1–7). N.D., not detected. More than 500 cells were counted for each independent experiment. (e) Detection of mutations at *EgGSL2* target sites at 72 h after electroporation by the T7EI assay. M, DNA ladder; arrowheads indicate bands of the digested PCR fragments. (f) Alignment of representative mutation patterns detected in *EgGSL2* target 1 (upper) or target 2 (lower) for Cas9 RNP‐treated samples at 72 h after electroporation and the wild‐type *EgGSL2* sequence. Ins/Del indicates the number of insertion/deletion bases. (g) Mutation (indel) frequencies of *EgGSL2* target 1 (left), target 2 (right) for Cas9 RNP‐treated and nontreated (‐RNPs) samples at 72 h after electroporation estimated by deep amplicon sequencing. Graphs represent the results of three independent experiments (Exp. 1–3) assessed by using the Cas‐Analyzer software (analysis parameters of nuclease type: single nuclease, select nuclease: SpCas9, comparison range: 70, minimum frequency: 0, WT marker range: 5). WT or Sub. indicates wild type or substitution. (h) Detection of the truncated PCR fragment at 72 h after the simultaneous introduction of the targets 1 and 2, in the *EgGSL2* gene. M, DNA ladder; arrowheads indicate bands of the truncated PCR product. (i) Alignment of representative mutation patterns in truncated PCR fragments and wild‐type *EgGSL2*. Ins/Del indicates the number of inserted/deleted bases. (j) Schematic illustration of designed ssODN and *EgGSL2* target site 2. (k) Detection of the knock‐in events in the *EgGSL2*‐targeting site at 72 h after co‐delivery of the *EgGSL2* target 2 Cas9 RNPs with ssODNs by restriction fragment length polymorphism. M, DNA ladder; arrowheads indicate bands of the *Eco*RI‐, *Eco*RV‐ or *Bam*HI‐digested PCR fragments. (l) Alignment of knock‐in patterns detected in *EgGSL2* target 2 Cas9 RNPs and ssODN‐treated samples at 72 h after electroporation and the wild‐type *EgGSL2* sequence.

To further advance Cas9 RNP‐based genome editing in *E. gracilis*, we demonstrated a precise ssODN‐mediated knock‐in experiment. We used the *EgGSL2* target 2 Cas9 RNPs with ssODNs (1 μL of 200 μm stock solution, final concentration was 4 μm in electroporation solution) that include 50 nt upstream and downstream of the *EgGSL2* target 2 cleavage site as homology arms and a 42nt knock‐in DNA fragment containing *Eco*RI, *Eco*RV and *Bam*HI sites (Figure 1j). We detected the effective knock‐in events in ssODN‐treated *E. gracilis* samples by digestion with these restriction enzymes (Figure 1k). We also performed Sanger sequencing of the knock‐in target region using total *E. gracilis* cells at 72 h after electroporation and identified 24 out of 35 randomly cloned PCR products that had a precise knock‐in sequence derived from our designed ssODN in the target site (Figure 1l). These results demonstrate that ssODN‐mediated knock‐in experiments are sufficiently applicable to *E. gracilis*.

Our results demonstrate, for the first time, transgene‐free targeted mutagenesis in *E. gracilis*, with an extremely high efficiency compared to that of previously reported genome editing attempts in other microalgae. We found maximum mutagenesis rates of more than 80% for *EgGSL2* targeting (Figure 1g), which are significantly higher than the rates for previously reported DNA‐free targeted mutagenesis using Cas9 RNPs (~1%) or Cpf‐1 RNPs with ssODN (~10%) in *Chlamydomonas reinhardtii* (Jeon *et al*., 2017; Spicer and Molnar, 2018), suggesting high editability of *E. gracilis* using RNP‐based methods. We hypothesized that the lower genome editing efficiency in microalgae from the Viridiplantae subkingdom is related to their unique cell walls or cell surface structures, which potentially inhibit RNP penetration into cells (Jeon *et al*., 2017), compared to photosynthetic unicellular eukaryotes lacking cell walls, such as *Euglena* in the phylum Euglenozoa. In *Trypanosoma cruzi*, a parasitic protozoan belonging to Euglenozoa, Medeiros *et al*. recently demonstrated highly efficient genome editing with a small SaCas9 (124 kDa), instead of the commonly used SpCas9 (163 kDa) (Medeiros *et al*., 2017), suggesting that the efficiency of RNP‐based genome editing is influenced by cell surface structures, as well as the physicochemical properties of RNP molecules. A high mutagenesis efficiency in the *E. gracilis* genome will accelerate its industrial use for foods and feeds. Using two gRNAs designed from adjacent sites on a chromosome, we also demonstrated the induction of a relatively large deletion (Figure 1i), which is useful for analysing the functions of non‐protein‐coding genetic codes such as long noncoding RNA genes and regulatory elements. We also demonstrated efficient knock‐in using Cas9 RNPs with ssODNs as DNA donors (Figure 1j–l). Although it is necessary to investigate the effects of knock‐in length and target site on the knock‐in efficiency in a future work, this technique will lead to more precise and elaborate genome editing, as well as the creation of stable knock‐in transformants for the elucidation of gene functions in *E. gracilis*.

The CRISPR‐based technology will significantly accelerate gene discovery and molecular breeding in *Euglena*. Using our RNP‐based genome editing approach in *E. gracilis*, we may be able to implement various CRISPR‐derived technologies, such as CRISPR‐mediated base editing and chromosome visualization (Doudna and Charpentier, 2014). CRISPR‐based gene targeting also enables us to introduce functional mutations into duplicated genes in *E. gracilis*, which is thought to be a polyploid (Yamada *et al*., 2016), similar to polyploid crop plants such as wheat and cotton. Recent examples of improved lipid productivity in *E. gracilis* by ion‐beam mutagenesis (Yamada *et al*., 2016) and RNA interference‐based down‐regulation of cellular signalling pathways (Kimura and Ishikawa, 2018) suggest that the targeting of negative regulator genes is a promising approach to identify genetic factors involved in lipid productivity in *E. gracilis* and to stably improve this trait. Our RNP‐based genome editing method in *E. gracilis* also provides a breakthrough to elucidate the functions of genes in other euglenids, the secondary plastid‐containing group in the Excavata supergroup, and opens up new avenues for improving their industrially important traits to promote sustainability by bio‐based material production.

## Competing interests

The authors declare that they have no competing interests.

## Authors’ contributions

TN conceived the study and designed the experiments. TN and YU performed experiments and analysed the data. KI performed the bioinformatics analysis. KY, OI and KS provided the biological materials. KM supervised the project, and TN and KM wrote the manuscript. All authors read and approved the final manuscript.

## References

[pbi13174-bib-0001] Doudna, J.A. , Charpentier, E. (2014) Genome editing. The new frontier of genome engineering with CRISPR‐Cas9. Science (New York, N.Y.), 346, 1258096. 25430774 10.1126/science.1258096

[pbi13174-bib-0002] Inui, H. , Ishikawa, T. and Tamoi, M. (2017) wax ester fermentation and its application for biofuel production. Adv. Exp. Med. Biol. 979, 269–283. 28429326 10.1007/978-3-319-54910-1_13

[pbi13174-bib-0003] Jeon, S. , Lim, J.M. , Lee, H.G. , Shin, S.E. , Kang, N.K. , Park, Y.I. , Oh, H.M. *et al*. (2017) Current status and perspectives of genome editing technology for microalgae. Biotechnol. Biofuels 10, 267. 29163669 10.1186/s13068-017-0957-z PMC5686953

[pbi13174-bib-0004] Kimura, M. and Ishikawa, T. (2018) Suppression of DYRK ortholog expression affects wax ester fermentation in *Euglena gracilis* . J. Appl. Phycol. 30, 367–373.

[pbi13174-bib-0005] Medeiros, L.C. , South, L. , Peng, D. , Bustamante, J.M. , Wang, W. , Bunkofske, M. , Perumal, N. *et al*. (2017) Rapid, selection‐free, high‐efficiency genome editing in protozoan parasites using CRISPR‐Cas9 ribonucleoproteins. MBio 8, e01788‐17. 29114029 10.1128/mBio.01788-17 PMC5676044

[pbi13174-bib-0006] Park, J. , Lim, K. , Kim, J.S. and Bae, S. (2017) Cas‐analyzer: an online tool for assessing genome editing results using NGS data. Bioinformatics (Oxford, England), 33, 286–288. 27559154 10.1093/bioinformatics/btw561 PMC5254075

[pbi13174-bib-0007] Spicer, A. and Molnar, A. (2018) Gene editing of microalgae: scientific progress and regulatory challenges in europe. Biology, 7, pii: E21. 10.3390/biology7010021 PMC5872047 29509719

[pbi13174-bib-0008] Suzuki, K. (2017) Large‐scale cultivation of euglena. In Euglena: Biochemistry, Cell and Molecular Biology ( Schwartzbach, S.D. , Shigeoka, S. , ed), pp. 285–293. Cham: Springer International Publishing.

[pbi13174-bib-0009] Tanaka, Y. , Ogawa, T. , Maruta, T. , Yoshida, Y. , Arakawa, K. and Ishikawa, T. (2017) Glucan synthase‐like 2 is indispensable for paramylon synthesis in *Euglena gracilis* . FEBS Lett. 591, 1360–1370. 28423179 10.1002/1873-3468.12659

[pbi13174-bib-0010] Yamada, K. , Suzuki, H. , Takeuchi, T. , Kazama, Y. , Mitra, S. , Abe, T. , Goda, K. *et al*. (2016) Efficient selective breeding of live oil‐rich *Euglena gracilis* with fluorescence‐activated cell sorting. Sci. Rep. 6, 26327. 27212384 10.1038/srep26327 PMC4876468

